# Improving home haemodialysis: Stability evaluation of routine clinical chemistry analytes in blood samples of haemodialysis patients

**DOI:** 10.11613/BM.2019.010709

**Published:** 2019-02-15

**Authors:** Lourens J.P. Nonkes, Maaike K. van Gelder, Hans Kemperman, Alferso C. Abrahams, Frans T.J. Boereboom, Maarten J. ten Berg, Karin G.F. Gerritsen

**Affiliations:** 1 Department of Clinical Chemistry and Haematology, University Medical Center Utrecht, Utrecht, The Netherlands; 2 Department of Nephrology and Hypertension, University Medical Center Utrecht, Utrecht, The Netherlands; 3 Diakonessenhuis, Utrecht, The Netherlands and Dianet Dialysis Center, Utrecht, The Netherlands

**Keywords:** haemodialysis, clinical chemistry tests, bicarbonates, blood specimen collection

## Abstract

**Introduction:**

A growing number of dialysis patients is treated with home haemodialysis. Our current pre-analytical protocols require patients to centrifuge the blood sample and transfer the plasma into a new tube at home. This procedure is prone to errors and precludes accurate bicarbonate measurement, required for determining dialysate bicarbonate concentration and maintaining acid-base status. We therefore evaluated whether cooled overnight storage of gel separated plasma is an acceptable alternative.

**Materials and methods:**

Venous blood of 34 haemodialysis patients was collected in 2 lithium heparin blood collection tubes with gel separator (LH PSTTM II, REF 367374; Becton Dickinson, New Jersey, USA). One tube was analysed directly for measurement of bicarbonate, potassium, calcium, phosphate, glucose, urea, lactate, aspartate aminotransferase (AST), and lactate dehydrogenase (LD); whereas the other was centrifuged and stored unopened at 4 °C and analysed 24 h later. To measure analyte stability after 24 h of storage, the mean difference was calculated and compared to the total allowable error (TEa) which was used as acceptance limit.

**Results:**

Potassium (Z = - 4.28, P < 0.001), phosphate (Z = - 3.26, P = 0.001), lactate (Z = - 5.11, P < 0.001) and AST (Z = - 2.71, P = 0.007) concentrations were higher, whereas glucose (Z = 4.00, P < 0.001) and LD (Z = 3.13, P = 0.002) showed a reduction. All mean differences were smaller than the TEa and thus not clinically relevant. Bicarbonate (Z = 0.69, P = 0.491), calcium (Z = - 0.23, P = 0.815) and urea (Z = 0.81, P =0.415) concentrations were stable.

**Conclusions:**

Our less complex, user-friendly pre-analytical procedure resulted in at least 24 h stability of analytes relevant for monitoring haemodialysis, including bicarbonate. This allows shipment and analysis the next day.

## Introduction

Home haemodialysis (HHD) attracts increasing attention as a viable dialysis option due to its association with improved clinical outcomes and reduced health care costs compared with in-centre haemodialysis (*1*-*3*). Dialysis in the home environment enhances patient’s freedom and autonomy and facilitates more frequent and/or prolonged haemodialysis sessions. A growing number of dialysis patients is treated with HHD.

Routine clinical chemistry testing is performed every 4 weeks to determine dialysis adequacy and for biochemical monitoring. In our HHD population, blood samples for this monthly routine testing are collected at home in lithium heparin (LH) gel tubes at the start and end of the dialysis session (*4*). Samples are centrifuged and subsequently plasma is transferred into a plain tube containing no anticoagulant by the patient, after which samples are transported to the hospital laboratory for analysis. This method precludes bicarbonate measurement as opening of the tube may result in falsely low bicarbonate concentrations due to CO_2_ dissipation (*5*, *6*). Therefore, patients are requested to have their blood drawn at a facility at least every 3 months for bicarbonate measurement, which precludes immediate pre- and post-dialysis bicarbonate measurement while these concentrations are important to know to prescribe the appropriate dialysate bicarbonate concentration (*4*). Both very low and high pre-dialysis plasma bicarbonate concentrations are associated with increased mortality in observational studies and can be corrected by adjusting the dialysate bicarbonate concentration and/or oral bicarbonate supplementation (*7*). Also, severe post-dialysis metabolic alkalosis should be avoided, since it is assumed to be associated with acute symptoms such as cardiac arrhythmia, muscle cramps, respiratory suppression and haemodynamic instability due to fast intradialytic decrease in plasma potassium and ionized calcium, and chronic detrimental effects such as vascular or metastatic calcifications, particularly in the presence of elevated calcium-phosphorus product (*8*, *9*). In addition, the current method is complicated for patients and therefore prone to errors.

This study was conducted to evaluate whether the use of plasma separator tubes (PST), which are left unopened after centrifugation, enables bicarbonate measurement in the home setting. The main advantage of a gel separator tube is that centrifugation of the tube separates the cell fraction from plasma by a gel layer, thereby minimizing time dependent changes in plasma analyte concentrations due to contact of the plasma with cellular components. In addition, it eliminates the need to transfer plasma into a different tube by patients, preventing exposure of plasma to air and avoiding contamination. Previous studies demonstrated the stability of a large range of analytes in gel separated plasma and serum during storage for up to 7 days, but little is known about the stability of bicarbonate in gel separated plasma during storage (*10*, *11*). We therefore measured bicarbonate in gel separated plasma after storage for 24 h and compared results with direct measurement. In addition, we studied the stability of other relevant analytes, which could be affected by cell dependent processes.

## Materials and methods

### Subjects

Venous blood specimens were collected from 34 patients on maintenance haemodialysis recruited from the haemodialysis clinic at the University Medical Center Utrecht, after providing informed consent. The study was performed in correspondence with the guidelines of the Helsinki Declaration on human experimentation, and local laws and regulations.

### Methods

Two 3.0 mL BD Vacutainer® LH blood collection tubes with gel separator (LH PST^TM^ II, REF 367374; Becton Dickinson, New Jersey, USA) were collected *per* patient from the vascular access and completely filled. After collection both tubes were centrifuged at 3913 RCF for 4 minutes at 4 °C using a Hettich Universal 320R (Hettich Benelux B.V., Geldermalsen, The Netherlands). One tube was directly analysed as part of routine analyses (0-h tube), whereas the other centrifuged tube was stored for 24 h at 4 °C without opening (24-h tube). After this latter period the plasma was transferred to a new tube that was centrifuged (3913 RCF for 4 minutes at 4 °C) and analysed directly afterwards. The following analytes were included in this study: bicarbonate (mmol/L), potassium (mmol/L), calcium (mmol/L), phosphate (mmol/L), glucose (mmol/L), urea (mmol/L), lactate (mmol/L), aspartate aminotransferase (AST, U/L), and lactate dehydrogenase (LD, U/L). All measurements were performed on an AU5811 routine chemistry analyzer (Beckman Coulter, Brea, California) and all samples were automatically checked for haemolysis (haemolysis-indices provided by the analyzer revealed no signs of haemolysis).

### Statistical analysis

As Kolmogorov-Smirnov testing for normality indicated that the obtained 0-h and 24-h tube data was not normally distributed, a two-sided Wilcoxon signed rank test was employed to test whether measured analyte concentrations where dissimilar between the 0-h and 24-h tube, *i.e.* whether there was an effect of 24h of storage on analyte stability. Additionally, the percentage deviation was calculated *per* pair of 0-h/24-h tubes (*i.e. per* patient) as follows: (t_24_ – t_0_)/t_0_ x 100. Here, t_0_ and t_24_ denotes the analyte measurement in the 0-h tube and 24-h tube, respectively. These values were used to compute the overall mean difference (%) per analyte. In addition, for each analyte, the mean difference was compared to the total allowable error (TEa = 0.25 x (CV_i_^2^ + CV_g_^2^)^0.5^ + 1.65 x 0.5 x CV_i_), as described by Ricos and colleagues (*12*). In this formula, CV_i_ denotes the within-subject coefficient of variation and CV_g_ the between-subject coefficient of variation. Thus, the TEa is based on the maximal allowable imprecision and bias and was used as an acceptance limit. If the mean difference of an analyte exceeded the TEa, this was considered a clinically relevant change. Accordingly, analytes were considered stable when the mean difference was smaller than the TEa. All statistical tests were performed in Matlab 2013 (Mathworks, Natick, USA).

## Results

Table 1 provides an overview of the values obtained from the 0-h and the 24-h tubes for each analyte, based on which the Z was calculated. Potassium (Z = - 4.28, P < 0.001), phosphate (Z = - 3.26, P = 0.001), lactate (Z = - 5.11, P < 0.001) and AST (Z = - 2.71, P = 0.007) concentrations were significantly higher after 24 h of storage at 4 °C (see Table 1). In contrast, glucose (Z = 4.00, P < 0.001) and LD (Z = 3.13, P = 0.002) were significantly lower. No significant changes were observed in bicarbonate (Z = 0.69, P = 0.491), calcium (Z = - 0.23, P = 0.815) and urea (Z = 0.81, P = 0.415) concentrations. For all measured parameters, the mean difference was smaller than their respective TEa values (see Table 1).

**Table 1:** Descriptive analysis of all measurements at 0 and 24 h

**Analyte**	**T_0_** **(N = 34)**	**T_24_** **(N = 34)**	**P** **(T_0_*vs*. T_24_)**	**Difference (%)**	**TEa** **(%)**
Bicarbonate, mmol/L	20.9 (19.8 - 22.2)	20.8 (19.9 - 21.7)	0.491	- 0.56 ± 4.69	5.6
Potassium, mmol/L	4.7 (4.2 - 5.1)	4.9 (4.4 - 5.3)	< 0.001	2.59 ± 2.63	5.61
Calcium, mmol/L	2.33 (2.25 - 2.41)	2.33 (2.26 - 2.40)	0.815	- 0.03 ± 1.66	2.55
Phosphate, mmol/L	1.37 (1.15 - 1.71)	1.39 (1.19 - 1.78)	0.001	2.45 ± 4.36	10.11
Glucose, mmol/L	6.3 (5.3 - 10.0)	6.2 (5.2 - 10.0)	< 0.001	- 2.12 ± 2.29	5.5
Urea, mmol/L	24.4 (19.6 - 27.9)	24.8 (19.0 - 27.8)	0.415	- 0.32 ± 3.05	15.55
Lactate, mmol/L	1.7 (1.3 - 2.4)	2.0 (1.6 - 2.7)	< 0.001	18.38 ± 12.31	30.4
AST, U/L	21 (15 - 28)	21 (15 - 30)	0.007	3.51 ± 7.23	16.69
LD, U/L	182 (168 - 193)	173 (161 - 197)	0.002	- 3.51 ± 5.57	11.4
Concentrations and activities of analytes are presented as median and interquartile range. Difference is presented as mean ± standard deviation. TEa - total allowable error. Comparison of the concentrations in the 0-h and 24-h tubes were performed using the two-sided Wilcoxon signed rank test. P < 0.05 was considered statistically significant.					

## Discussion

The stability of nine plasma analytes, including bicarbonate, was demonstrated after storage for 24 h at 4 °C in an unopened PST. The change in concentrations during storage for all analytes did not exceed the acceptance limit for a clinically relevant change.

Importantly, bicarbonate concentrations were stable. This may improve the care of HHD patients, since it renders regular hospital visits for bicarbonate measurement unnecessary and enables direct measurement of pre- and post-dialysis bicarbonate concentrations, which allows adequate prescription of dialysate bicarbonate concentrations to avoid very low and high pre-dialysis plasma bicarbonate concentrations and post-dialysis metabolic alkalosis, which are associated with adverse outcome. The current blood collection method in the home setting does not allow for reliable bicarbonate determination, since opening of the tube allows for CO_2_ to dissipate from plasma into the atmosphere, resulting in a shift of the carbonic acid equilibrium to the left (towards CO_2_ generation, Equation 1), and thus, falsely lowering HCO_3_^-^ and increasing pH (*13*).

CO_2_ + H_2_O ↔ H_2_CO_3_ ↔ HCO_3_^-^ + H^+^ (Equation 1)

On the other hand, interference of cellular constituents with plasma during storage of unopened centrifuged gel separator tubes with our proposed novel pre-analytical procedure may theoretically also result in falsely decreased bicarbonate concentrations, since ongoing anaerobic metabolism by living cells (*e.g.* erythrocytes) leads to 1 mmol of H^+^ *per* mmol of lactate produced. This results in a shift of the carbonic acid equilibrium to the left (towards CO_2_ generation, Equation 1). In addition, carbonic anhydrase in the erythrocytes may rapidly convert CO_2_ to carbonic acid, which dissociates into H^+^ and HCO_3_^-^ (Equation 1). In the systemic circulation, the intracellular rise in bicarbonate concentration stimulates the exchange of bicarbonate with plasma chloride across the erythrocyte membrane (*14*). In theory, this process may also occur in the gel separator tube, falsely increasing plasma bicarbonate concentrations. In the present study, bicarbonate concentrations remained stable in unopened centrifuged gel separator tubes. This proves that tube integrity is maintained during storage, preventing dissipation of gases from plasma into the atmosphere. Also, it demonstrates the efficiency of the gel barrier in preventing interference of cellular constituents with plasma.

Two studies evaluated bicarbonate stability in gel separator tubes and found that bicarbonate concentrations decreased after 8-24 h of storage at 4 °C after exposure of the sample to air (*5*, *6*). Thus, it seems that keeping a tight seal during storage is crucial for bicarbonate stability.

The other analytes measured in this study were selected because they are prone to change by interference of the cell fraction with plasma. Loss of cell integrity during prolonged storage can result in leakage of intracellular components, which may diffuse across the gel barrier to the plasma site leading to erroneous results. The observed increase in phosphate, lactate, aspartate aminotransferase and potassium concentrations is in line with this and has also been described in previous studies after 24 h of storage using gel separator tubes, albeit inconsistently, as a significant increase was not always observed (*5*, *6*, *15*). For potassium inhibition of the sodium/potassium ATPase pump during cold storage, resulting in a net potassium efflux out of the cell, is also a likely contributor to the increased potassium concentration observed after 24 h. Unexpectedly, and in contrast to previous studies, LD concentrations were decreased (*5*, *6*, *15*). The origin of this effect is unclear. It may relate to the observation that certain LD isoforms are found labile during prolonged 4 °C storage, resulting in a decrease in total LD activity (*16*). In accordance with previous studies, glucose concentration was reduced after 24 h, which is probably due to the continuing glycolytic action of cells that causes a decrease in glucose and increase in lactate concentrations, the end-product of anaerobic glycolysis (*6*, *15*, *17*). Importantly, none of the above-described changes in analyte concentrations measured in this study exceeded the TEa and thus were not clinically relevant.

The strengths of our study include evidence of bicarbonate stability after storage for 24 h and definition of an acceptance limit for a clinically relevant change in analyte stability. This study is limited by the measurement of only a selection of analytes for the given study conditions and the fact that the draw order of the 0-h and 24-h tube was not systematically randomized. Since transport conditions were not studied, we advise to ship tubes in a portable cooler bag in upright position.

In conclusion, centrifuged lithium heparin blood collection tubes with gel separator can be stored unopened at 4 °C for at least 24 h without compromising analyte stability, including bicarbonate. These findings allow for the collection, centrifugation and storage of blood samples at the patient’s home without the need to transfer plasma into a new tube by the patient. Furthermore, shipment and analysis can be performed the next day.
